# On the Employment of Tartar Emetic to Relieve Rigidity of the Cervix and Os Uteri, in Cases of Parturition

**Published:** 1851-01

**Authors:** 


*On the Employment of Tartar Emetic to Relieve Rigidity of the Cervix and Os Uteri, in cases of Parturition.* By ARCHIBALD HALL, M. D., L. R. C. S. E., *Lecturer on Materia Medica, M.Gill. College; Consulting Physician to the Montreal General Hospital, and to the University Lying-in Hospital,* &c.

Of the various causes which tend to complicate a labor, and render it tedious, discouraging to the accoucheur, and harassing to the sufferer, few are of more common occurrence than rigidity of the os or cervix uteri—few, the removal of which is generally more readily effected, and in which timely relief obviates such unpleasant consequences.

The object of the present paper is not to propose a new method of relief under the circumstances mentioned, but to call attention to, and re-produce a means which has been strangely and inconsiderately neglected; which is not dwelt upon at all in most of our works of authority, or does not occupy that position which its merits so eminently deserve.

Rigidity of the os uteri consists in a deficient dilatation of the muscular fibres of that portion of the uterus, coupled with heat, dryness and tenderness, in proportion to the duration of time in which it has existed. If the rigidity has been of short duration, these latter phenomena will not be found to exist; but if of long duration, they will, by their presence, indicate the existence of local inflammation to a greater or less degree. This unequal or irregular contraction of the muscular fibres of the neck of the uterus, depends originally upon irritation of those fibres localy applied, which may, if not relieved, end in inflammation—which, in its turn, proves a secondary exciting cause; and in order, therefore, to obviate a serious complication in which might have been otherwise a perfectly natural labor, the earlier this source of difficulty is detected the bettter, as the more conducive to the practitioner's reputation, and the safety and comfort of the patient.

Pathologically examined, rigidity of the cervix uteri consists in a *spasmodic contraction* of the circular fibres of the locality specified, and is induced by causes of an irritant nature.* The cause most frequently in operation, is the early pressure of the child's head upon the whole cervix,

*Dr. W. Tyler Smith, describes the opening of the os uteri, as depending "partly upon the mechanical distension of the non-contractile tissue, and partly upon the muscular dilatation of the contractile fibres which enter into the composition of the os and cervix uteri;" and applying this fact to the explanation of rigidity of the os uteri, he shows that the latter consists either in the absence of distensibility, or of dilatability, or in both of these states combined. * * * "In numerous cases, both the muscular and mechanical forms of rigidity exist, and mechanical rigidity is itself sometimes a cause of spasmodic closure of the os uteri. The heat and irritability of the os uteri render it morbidly excitable, and the presence of the liquor amnii. or the presentation instead of exciting a reflex dilatation of the mouth of the uterus, excites it to spasmodic contraction."—*Braithwaite's Retrospect, Vol.* 19, *from Lancet, Nov* 25 1850.

"This unfavorable state of the os uteri, [rigidity] may be discovered to exist at the very commencement of its dilatation, or may not occur until the process of dilatation has somewhat advanced; in the former case it is the result of a premature rupture of the membranes, in the latter, most probably owing to a spasmodic contraction of the cervical fibres, produced by the irritation of unnecessary and too frequent vaginal examinations, or the effect of pressure on the cervix between the child's head and bony pelvis, &c., &c.,"—*Dr. A. Tyler's Lectures on Practice Obstetricy in British Record of Obstetric Medicines, Vol.* 2.

or a partial pressure exercised upon the anterior wall, operating upon that portion lying between the presenting part and the pubis. Under the latter circumstance, the spasm may be partial, involving only the anterior lip of the cervix; under the former circumstance, the spasmodic action may be complete, and in the majority of cases, will be induced by the early rupture of the membranes, in either an artificial or spasmodic manner, with a general pressure of the child's head, or other presenting portion, upon the cervix, thereby compressing it upon the brim of the pelvis.

Cases, however, are recorded in obstetric works, in which the rigidity has depended upon a *cartilaginous* degeneration of the muscular fibres of the cervix, and upon cicatrization, the consequence of sloughing from injuries which that part of the viscus may at some antecedent period have received. Such cases, fortunately very rare, require a specific line of treatment by the knife. The fact is here only worthy of notice as having originated a division of this peculiar complication of labor into the two varieties of *symptomatic* and *idiopathic.*

Flowing from the idea, perfectly correct, that the irritation is likely to be succeeded by inflammation, unless quickly relieved, most obstetric works recommend, to overcome the difficulty, the practice of venesection, or the exhibition of opium.* But few authors advise a resort to tartar

---

*When the orifice is rigid, undilated and undilatable, we should employ to overcome this resistance, the various msans I have already suggested; bleeding if the patient be phlethoric, baths, unctions on the cervix, with the extract of belladonna."—*Chailly's Midwifery, Am. Ed.* 1844, *page* 273.

"When there is much vascular excitement, &c., it is almost always advisable to abstract blood. The quantity must, of course, be regulated by circumstances; but as a general rule, I would say, let it be the minimum required. I think I have observed considerable benefit to result in such cases as these, from the employment of nauseating doses of tartar emetic, in conjunction with, or as a substitute for bleeding; but there certainly are circumstances under which the latter cannot safely be dispensed with. Opium has been much recommended as a relaxant, but it is a medicine, the effect in particular, we cannot accurately measure. . . . . . . . Other means, as fomentations, the introduction of tallow into the vagina, application of belladonna to the os uteri, injections of tobacco, the warm bath, &c., have been recommended; but of their effects I know nothing from experience, and *a priori* see no reasons that can sanction their use."—*The Dublin Practice of Midwifery, by H. Maunsell, M. D.*, 1845. *Page* 142 *and seq.*

"Nauseating remedies, and even tobacco injections have been tried to a considerable extent, for the purpose of relaxing the mouth of the uterus, but they produce little or no good effects, and cause much suffering to the patient."—*Library of Medicine, Vol.* 6, *page* 199.

"For myself, in endeavoring to effect a relaxation of the soft parts, fomentations and bleeding are the remedies to which I principally confide."—*Principles and practice of Obstetric Medicine, by J. Blundell, M. D.*

"For rest of body, tranquility of mind, the abstraction of stimuli, *the loss of blood,* free bowels, and not allowing the soft parts to be disturbed by ill-timed and officious touching, or ill-conceived manual aid at the mouth of the uterus, have, in a thousand instances. overcome every difficulty presented by simple rigidity."—*Dewee's system of Midwifery*, 1847, *page* 302.

"If the patient be strong, phlethoric, and disposed to make violent straining efforts, a free depletion from the arm will be of use; it diminishes the tendency to inflammation, and produces a feeling of exhaustion in the patient which induces her to bear her pains more patiently. In order to produce such an effect, depletion may be followed by tartarized antimony, in small doses, so as to excite nausea, &c."—*Lectures on Natural and difficult parturition, by E. W. Murphy, M. D.*, 1846, *page* 128.

The multiplication of authorities is, I think, unnecessary.

emetic; and in most of the works of authority to which I have had access, they recommend it as an *adjuvant* to the venesection, and by way of securing the effects of the bleeding upon the system. The local application of belladonna has also been advised by Mr. Dubois of Paris.†

Therapeutic agents are of value exactly in accordance with the effects which, under peculiar circumstances, they are capable of producing. This is apprehended to be an axiom in medicine, and by which the relative value of medical agents may be accurately measured. I propose now to examine these several modes of treatment, keeping the above axiom in view.

1. With regard to venesection. This seems to be the established rule of practice, and is sanctioned by every author of note. Bleeding, in its influence upon the system, is one of the most certain and powerful sedatives which we possess, and presents strong claims to our consideration, under the peculiar circumstances of the case. To be of any real value, however, it requires to be vigorously practiced, and relaxation will be found rarely to follow, until a large quantity of blood has been extracted, and syncope is on the point of supervention. There can be no question that the best effects follow its employment, as far at least as rigidity is concerned. But is this line of practice always expedient? Is it always imperiously demanded? When the os uteri exhibits heat, dryness and tenderness, such effects being the evidence of existing inflammation, the propriety of the practice cannot be questioned. But before the development of these symptoms, several hours may elapse, during which the effects of irritation alone are apparent as indicated by the mere rigidity. Under these circumstances, bleeding *is not* imperiously demanded, chiefly because it entails an unnecessary withdrawal of blood, inducive of debility, and protracted recovery—thus effecting more than we desire. The practice, however, is recommended and sanctioned by Burns, Dewees, Blundell, Ramsbotham, Chailly, Cazeaux, and a host of others.

2. The exhibition of opium is not always attended with the advantages which we might *a priori* be led to expect from its well known narcotic powers. If exhibited, full doses should be employed; but as in the case of bleeding, it is liable to effect too much—it may lull the uterine contractions, which it is generally desirable to expedite.

3. The local application of Belladonna. This was first proposed by Chaussier, who suggested its use in the form of ointment made with cerate, reasoning analogically from the influence of this medicine upon the iris. Dubois subsequently used the extract in its undiluted state. The practice is peculiarly French, and has not been followed to any extent by either British or American accoucheurs. That the application of the belladonna will produce relaxation, is admitted on all hands; but the extent of that relaxation cannot be predetermined. It may affect the whole muscular coat of the uterus, and be thus productive of alarming consequences.

4. Tartar Emetic. The prostration and muscular relaxation produced by this agent, almost naturally indicate its employment in cases of the

†Besides the employment of bleeding, tartar emetic, opium, and belladonna, advantage has also been derived from the local application of leeches, the warm bath, the exhibition of purgatives and artificial dilatation. More lately, Dr. Scanzoni advises a continued douche of warm water applied to the os uteri by a special aparatus; and, more lately still, Dr. Snow has found advantage from the employment of chloroform.

kind we are considering. Nausea having been once established, the rigidity will in a very large majority of cases be found readily to yield. Tartar emetic seems almost to exert a special influence on the cervix, for while the contractions of the fundus and body of the uterus are not interfered with, dilatation of the cervix will be found to proceed rapidly, and this the result of the re-establishment of the reflex actions existing between the stomach and the uterus, which are apparently suspended.

The few authors who have advocated the employment of tartar emetic in these cases, have generally prescribed it in doses of one-fourth of a grain repeated every three or four hours, until its influence became apparent by the gradual dilatation of the mouth of the uterus. There is certainly no worse system of midwifery than the meddlesome midwifery; but if, in any case, interference is demanded, whether of a manual or medicinal nature, the object should be a speedy delivery, consistent with the safety of the mother and the child. My own observation has led me to the belief that it may be safely resorted to in much larger doses, and with more prompt relief; and I cannot but view the mode of employing the medicine indicated above, as attended with a very considerable and quite unnecessary sacrifice of time on the part of the accoucheur, and of prolonged suffering on the part of the patient. I accordingly, prescribe it in one grain doses, repeated every half hour, and I have not as yet, found it necessary to exhibit the medicine in such doses more than thrice, relaxation most commonly following the exhibition of the first grain. The following cases, from rough notes in my possession will, I hope, demonstrate the value of the practice:

Case 1.—March 2, 1846. Mrs. P., (Bonaventure Street,) in labor of her first child; had been under the care of a midwife for the last seven or eight hours. Her age was 36, stout and healthy; was called about 1 a. m.; for the last two or three hours labor had made no progress; pains severe and regular; membranes ruptured about 9 p. m., the preceding evening. On examination, the perineum and vagina were found sufficiently soft and yielding; the os uteri was dilated to about the size of a half crown, was thick, firm and rigid, through which the scalp protruded to a considerable extent. The impediment being clearly the rigidity of the os uteri, I prescribed to her one grain of tartar until relaxation followed; after three doses were taken, relaxation followed, and the child was born in the course of an hour afterwards. In this case no nausea supervened until the second dose had been swallowed, and vomiting did not occur until the third had been administered. The vomiting was not urgent, and did not appear in the slightest degree impede the progress of the labor. The subsequent recovery was complete.

Case 2.—Dec. 20, 1846. Mrs. L., (Notre Dame Street.) Symptoms of labor came on about 11 p. m., and I was sent for about 3 a. m. This lady was of a delicate constitution, and had already given birth to two children, both of whom were still-born. Of the cause of this I coul d not satisfy myself at the time, but in both instances the labors were protracted. On examination, I found the membranes protruding through a partially dilated orifice, and could with some difficulty satisfy myself of a natural presentation. In the course of about two hours the membranes ruptured, and the child's head having descended lower, the presentation was accurately determined, left occipito-cotyloid. Every thing appearing to progress favorably, no further examination was effected for an hour; at this period, the anterior lip of the os uteri was found forced down in front of the head during each pain, acting at the same time as a tight band across it. Several ineffectual attempts were now made to push it up during the pains; these proving abortive, recourse was had to the tartar emetic. Half a grain was immediately exhibited in a little water, and before the expiration of half an hour, the rigidity of these fibres of the cervix had yielded, the child's head was firmly forced down, and at about nine o'clock a, m., she gave birth to a living child. It is not an improbability that the two previous protracted labors of this lady were due to the same cause, and that interference was too long postponed. I have attended this lady a second time since, and the same anomalous condition of the cervix uteri occurred.

Case 3.—June 3, 1847. The husband of Mrs. O'N. (St. Joseph Street) requested me to visit his wife, who had been about thirty-six hours in labor. I found her under the charge of an ignorant woman, who claimed to act as a midwife. She was about 40 years old, had had six children previously, was of short stature, and very muscular. The membranes had ruptured, and the waters had dribbled away several hours previous to my visit; the head rested upon the brim of the pelvis; os uteri dilated to the size of a crown, hot and irritable, and feeling like a ligature applied over the presenting parts; the vagina hot and tender, and the labia considerably swollen. Found that the midwife had examined her very frequently, and had caused some pain during her manipulations. These effects might have been ascribed as much therefore to improper interference, as to the pressure of the head on the bony pelvis, compressing the cervix. Inflammation was here clearly setting in, and I abstracted about ℥xxx of blood; she bore it well; but in the course of about 20 minutes, upon examination, finding not the least impression upon the os uteri, I gave her the tartar emetic, in preference to further abstraction of blood. After three grains had been taken, the rigidity yielded, and although the perineum, at a subsequent period, offered an obstacle, yet patience overcame the difficulty, and the child was born, although dead.

Case 4.—(Nov. 5, 1850.) Mrs. M'P., (Cheneville Street,) had been under the care of one of the most experienced midwives in the city. Her labor up to 7 o'clock, p. m., had lasted for nearly forty-eight hours, and was, in all other respects, perfectly natural. I was sent for at that hour; it was her first child. The mother was healthy, stout, and well formed, and aged about twenty-five. On examination every thing was normal, with the exception of a rigid band of the os uteri, dilated to about the size of a dollar, encircling the presenting vertex. The membranes had ruptured several hours previously, and by stethoscope, the fœtal pulsations were clearly audible. In this case, one grain of tartar emetic produced such immediate and complete relaxation, that the child was born in about thirty-five minutes afterwards. In no case which has come under my observation have I witnessed such marked beneficial results following its employment.

It must be admitted, however, that cases may be met with in which its exhibition would be decidedly improper. These exceptions to the rule will be found to occur in women of delicate habit and leucophlegmatic temperament. They are cases in which the action of the remedy, if exhibited, may proceed too far; which, unable to resist the prostrating effects of the medicine, might be followed by a collapse, to which the vital powers of the system might succumb. This is a contingency which should be sedulously kept in view; and prudence demands, therefore, some care in the selection of proper cases for its exhibition.

I have selected the above cases, and have given them as abstracts from my note book, for the purpose of illustrating the effects of the medicine, and of drawing more general attention to it. I place them before the profession with that object alone in view.

Montreal, Nov. 15, 1850.

*British American Med. and Phys. Jour.*

